# Predicting Speech Intelligibility Decline in Amyotrophic Lateral Sclerosis Based on the Deterioration of Individual Speech Subsystems

**DOI:** 10.1371/journal.pone.0154971

**Published:** 2016-05-05

**Authors:** Panying Rong, Yana Yunusova, Jun Wang, Lorne Zinman, Gary L. Pattee, James D. Berry, Bridget Perry, Jordan R. Green

**Affiliations:** 1 Department of Communication Sciences and Disorders, MGH Institute of Health Professions, Boston, Massachusetts, United States of America; 2 Department of Speech-Language Pathology, University of Toronto, Toronto, ON, Canada; 3 Department of Bioengineering, University of Texas at Dallas, Richardson, Texas, United States of America; 4 Callier Center for Communication Disorders, University of Texas at Dallas, Richardson, Texas, United States of America; 5 Sunnybrook Health Sciences Centre, Toronto, ON, Canada; 6 Neurology Associates, P.C., Lincoln, Nebraska, United States of America; 7 Massachusetts General Hospital, Boston, Massachusetts, United States of America; University of Ulm, GERMANY

## Abstract

**Purpose:**

To determine the mechanisms of speech intelligibility impairment due to neurologic impairments, intelligibility decline was modeled as a function of co-occurring changes in the articulatory, resonatory, phonatory, and respiratory subsystems.

**Method:**

Sixty-six individuals diagnosed with amyotrophic lateral sclerosis (ALS) were studied longitudinally. The disease-related changes in articulatory, resonatory, phonatory, and respiratory subsystems were quantified using multiple instrumental measures, which were subjected to a principal component analysis and mixed effects models to derive a set of speech subsystem predictors. A stepwise approach was used to select the best set of subsystem predictors to model the overall decline in intelligibility.

**Results:**

Intelligibility was modeled as a function of five predictors that corresponded to velocities of lip and jaw movements (articulatory), number of syllable repetitions in the alternating motion rate task (articulatory), nasal airflow (resonatory), maximum fundamental frequency (phonatory), and speech pauses (respiratory). The model accounted for 95.6% of the variance in intelligibility, among which the articulatory predictors showed the most substantial independent contribution (57.7%).

**Conclusion:**

Articulatory impairments characterized by reduced velocities of lip and jaw movements and resonatory impairments characterized by increased nasal airflow served as the subsystem predictors of the longitudinal decline of speech intelligibility in ALS. Declines in maximum performance tasks such as the alternating motion rate preceded declines in intelligibility, thus serving as early predictors of bulbar dysfunction. Following the rapid decline in speech intelligibility, a precipitous decline in maximum performance tasks subsequently occurred.

## Introduction

Speech is produced through the coordinated actions of the articulatory, resonatory, phonatory, and respiratory subsystems. Impairments in one or more of these speech subsystems can compromise speech intelligibility [1,2]. Modeling of the separate and combined impact of subsystem impairments on speech intelligibility has been a significant empirical challenge, but is important for improving speech motor assessments and identifying speech treatment targets [3,4].

Despite the need for exploring the impact of subsystem impairments on speech intelligibility, only a few studies have attempted modeling the effect of multiple subsystems on speech decline [5,6]. These studies used auditory-perceptual features of different speech dimensions (i.e., voice quality, articulation, nasality, and prosody) [5] or acoustic measures indicative of the integrity of subsystem function (e.g., F2 slope, vowel space, fundamental frequency [F0], and nasal resonance) [6] as predictors of speech intelligibility, which accounted for over 80% of the variance in speech intelligibility across various dysarthric populations.

Models of dysarthric speech such as the one developed by De Bodt et al. [5] based on auditory-perceptual scales are important for understanding the perceptually salient features that drive intelligibility decline and for developing listener-based strategies to enhance intelligibility [7,8]. Models based on auditory-perceptual ratings, however, are challenged by listener biases making it difficult to determine how affected was driven by differences among listeners performing the perceptual assessments rather than differences among the speech profiles of the speakers [9]. In contrast, models on the basis of acoustic features, such as the one developed by Lee et al. [6], are based on quantifiable markers of speech performance. Acoustic parameters, however, do not unambiguously represent the status of individual speech subsystems. An improved subsystem-based intelligibility model may, therefore, require the inclusion of physiologic-based indices of speech subsystem performance where possible.

A robust intelligibility model also requires a sufficient sample of participants who exhibit varying degrees of impairment across different subsystems. The population with dysarthria due to amyotrophic lateral sclerosis (ALS)–a motor neuron disease–meets this criteria because the disease often differentially impairs the articulatory, resonatory, phonatory, and respiratory speech subsystems, and the overall speech performance declines progressively over time [1,2,10,11,12,13].

Over the past few decades, instrumentation-based measures have been used to characterize the decline across speech subsystems due to ALS [3,11,12,14,15,16,17,18]. These studies were mostly based on single speech subsystems and have identified a set of candidate measures that showed sensitivity for detecting early changes in speech subsystem performance [2,3,19]. For example, Yunusova et al. [20] reported associations between the decline in speech intelligibility and changes in the extent, speed, and duration of lip and jaw motions. Although these findings suggested contributions of individual subsystems to speech intelligibility decline, measures of a single subsystem are unlikely to sufficiently account for the variance in the longitudinal decline of speech intelligibility in ALS [11,12]. Therefore, the primary goal of this study was to determine the collective and individual contributions of speech subsystem impairments to the longitudinal decline of speech intelligibility in persons with a range of severities of ALS.

To achieve this goal, we employed a robust statistical modeling approach to overcome several prior barriers to developing an explanatory model of intelligibility decline. First, although the variables that best capture speech subsystem decline are unknown, most prior studies have been based on a priori assumptions about the salient physiologic variables that drive speech intelligibility decline. In this study, we screened a large number of subsystem variables using a data-driven approach, which produced an optimized set of speech subsystem variables for predicting speech intelligibility decline. The second limitation of prior studies lies in the assumption of a linear association between intelligibility decline and subsystem involvement. Research on the longitudinal decline of speech intelligibility in ALS has uniformly demonstrated that the rate of intelligibility decline is not consistent during disease progression [3,19]. Specifically, the longitudinal decline of intelligibility can be described as approximately bi-phasic: (1) during the early phase, intelligibility remains relatively high and declines at a slow rate, and (2) during the late phase, intelligibility declines rapidly as the disease progresses, resulting in the eventual loss of speech communication within a relatively short time span [3,19]. To model the bi-phasic trajectory of intelligibility decline, a more sophisticated nonlinear model is required. In this study, we compared linear models with bi-phasic nonlinear models to determine the best-fitting model of intelligibility. In addition, unlike previous studies that used regression analyses, we used mixed effects models to account for the heterogeneity among different individuals. This modeling approach provides a robust means to model the group pattern of speech intelligibility decline while taken into account the variability across individuals.

Using the data-driven and statistical modeling approaches, this study addressed two aims: (1) to identify subsystem variables related to speech intelligibility impairment in patients with ALS, and (2) to determine the relative contribution of the subsystems to the overall speech intelligibility decline over the course of the disease.

## Materials and Methods

### Participants

The study was approved by the Ethics Research Boards at the Sunnybrook Research Institute in Toronto, University of Nebraska–Lincoln, and the MGH Institute of Health Professions. Sixty six participants (37 males and 29 females) aged from 39 to 79 years old (M = 57 years, SD = 10 years) took part in this study. All participants were diagnosed with possible, probable or definite ALS by a neurologist (Authors 5&6), who recruited them into the study. All consecutive patients were invited to participate and everyone who was able and willing to participate in a longitudinal study was recruited and consented in a written form. Participants were excluded if they were unable to speak English fluently, read at the grade 5 level, reported hearing impairment, or reported significant visual impairment preventing them from being able to read. We also excluded those who showed signs of cognitive impairment as measured by the Montreal Cognitive Assessment (MoCA; cut of score <26; [21]). None of the patients were on medications known to affect speech production [22].

The characteristics of the participants were summarized in S1 Table. Among all participants, 15 reported bulbar onset, 41 spinal onset, 6 mixed bulbar and spinal onsets, and 4 had an unknown onset site. Disease duration varied among participants. At the first session of the study, patients were on average 12 months post diagnosis (SD = 18 months). The severity of ALS and its bulbar presentation also varied among participants, as assessed by the ALS Functional Rating Scale–Revised (ALSFRS-R). The ALSFRS-R score (0~48) was obtained from 12 survey questions that assess the degree of functional impairment with the score of each question ranging from 4 –least impaired to 0 –most impaired. The ALSFRS-R scores of the 66 participants in this study ranged between 29 and 48 at the first session, with a mean of 38 and SD of 5. The bulbar subscore, estimated based on the first 3 questions of the scale, assessed the bulbar function with a maximum score of 12 and ranged between 4 and 12, with a mean of 10 and SD of 2 in our sample.

All participants were recorded longitudinally over multiple sessions; the duration between the first and last sessions ranged from 42 days to 1798 days (M = 455 days, SD = 365 days). Patients were scheduled for follow-up visits to coincide with clinical visits, approximately every three months. Ultimately, inter-visit durations varied substantially, in part dependent upon the rate of disease progression. The average number of sessions across the participants was 7 (SD = 5). As expected, study retention was challenging due to immobility from ALS progression [23,24], which resulted in a number of missing data throughout the recordings.

### Data acquisition

#### Measures of speech subsystems

The speech functions of the articulatory, resonatory, phonatory, and respiratory subsystems were assessed during multiple speech tasks using a variety of acoustic, aerodynamic, and kinematic instruments. A brief description of the instrumentation, acquisition settings and measurements is in S2 Table; more detailed descriptions have been published previously in Green et al. [3] and Yunusova et al. [25]. Briefly here, a 45-minute recording protocol comprised of acoustic, aerodynamic, and kinematic methodologies was used to monitor performance across subsystems during each visit.

A total of 58 measures were collected across all four subsystems and comprised a multi-factorial database. Collecting a substantial number of measures and implementing a variable discovery process was necessary because the variables that best capture the decline in speech performance with disease progression of each subsystem are unknown.

#### System-level speech measurement

In addition to the subsystem measurements, the Sentence Intelligibility Test (SIT; [26]) was performed to obtain the system-level measurements of speech intelligibility and speaking rate. As a standard clinical approach to assess speech intelligibility in persons with motor speech disorders, SIT has been previously used to index the severity of bulbar ALS in multiple studies [3,19,20,27].

During the test, participants were asked to read a list of 10 sentences of varying length (from 5 to 15 words) randomly generated by the SIT software. The speech samples of each participant were transcribed by one naive listener who was unfamiliar with either the test materials or the dysarthric patterns of the participants. Based on the SIT, speech intelligibility (i.e., the percent of words correctly transcribed out of the total number of words) and speaking rate (i.e., the number of words read per minute) were calculated automatically by the SIT software.

### Data reduction

**Fig 1:**
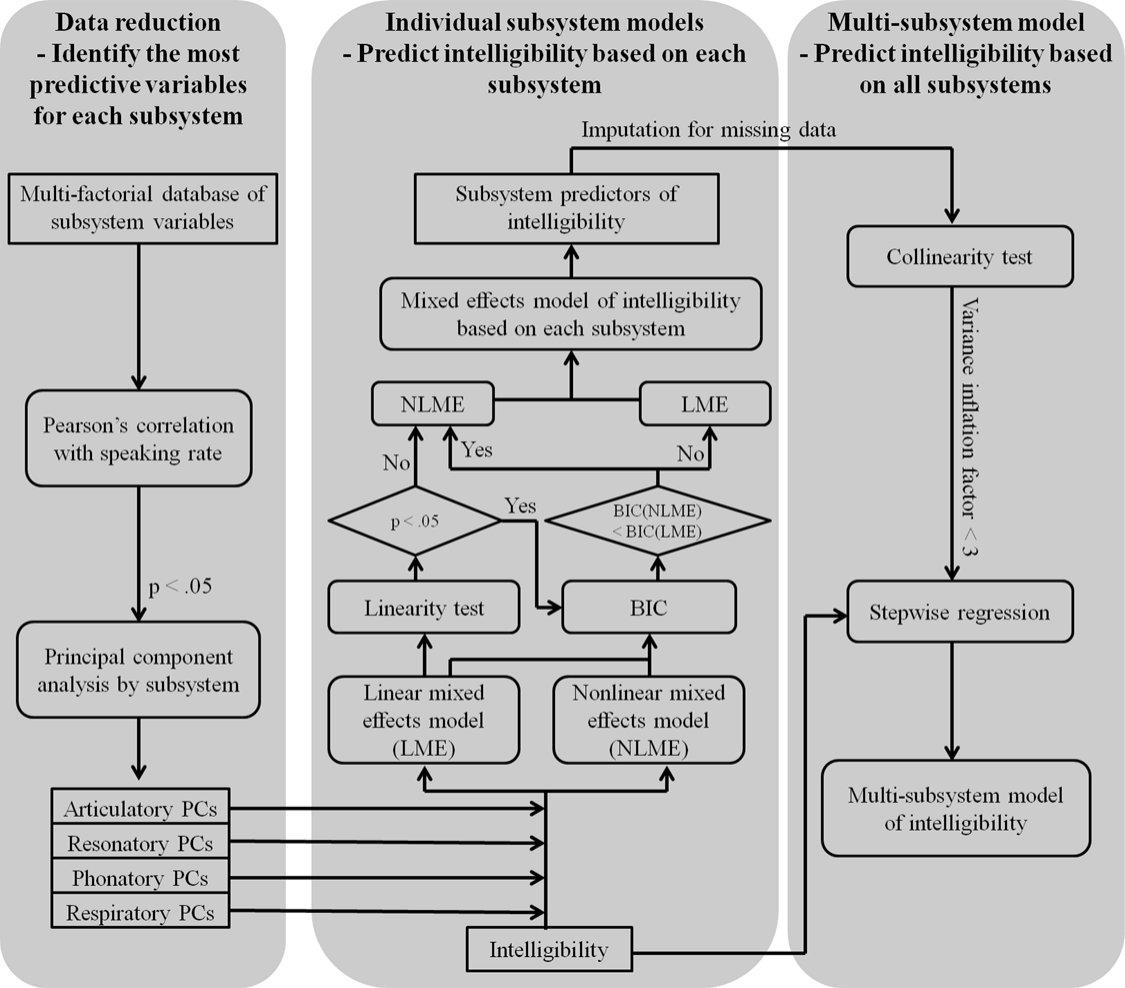
Flow chart for (1) the variable selection and dimensionality reduction for each subsystem; (2) constructing of individual subsystem models of speech intelligibility; and (3) development of a multi-subsystem model of speech intelligibility.

We performed data reduction combining Pearson’s correlation and principal component analysis (PCA) following the steps displayed in the first panel of Fig 1. The first step was a variable selection process, which identified the variables that were sensitive to bulbar decline by calculating their correlations with speaking rate using Pearson’s correlation coefficients. While there is currently no gold-standard measure of bulbar disease severity for ALS, speaking rate was chosen as an indicator of bulbar decline because, unlike speech intelligibility, it is known to decline earlier and at a relatively constant rate [19]. The correlation analysis identified 25 variables (see S3 Table) that were significantly correlated (*p* < .05) with speaking rate. Specifically, articulatory subsystem was represented by 12 variables that represented the maximum and minimum velocities of the lips and jaw during “Buy Bobby a puppy”, and the number of repetitions, duration, and rate of the AMR task. Resonatory subsystem was represented by six variables that represented the velopharyngeal aerodynamics (e.g., oral and nasal airflow) during contrastive syllables /ma/ and /pa/, the time lag between /m/ and /p/ in “hamper”, and the nasalance during sentence reading. Phonatory subsystem was represented by two variables that corresponded to maximum fundamental frequency (F0) and average laryngeal airway resistance. Respiratory subsystem was represented by five variables that quantified speech pausing patterns. All of the subsystems’ variables served as the input for the second step of the analysis.

In the second step, the selected variables of each subsystem were subjected to a principal component analysis [28] to further reduce the dimensionality of the dataset and eliminate collinearity (Fig 1). PCA is a statistical procedure that uses an orthogonal transformation to convert a set of observations of possibly correlated variables into a set of linearly uncorrelated principal components [29]. Each principal component was represented as a weighted sum of the subsystem variables, where the variables that determined the primary speech performance of the subsystem were assigned high weights. For each subsystem, a minimum set of principal components that jointly accounted for over 90% of the total variance was selected to serve as the candidate predictors of speech intelligibility loss.

### Modeling of speech intelligibility decline

To determine the relative contribution of each subsystem to the overall speech intelligibility decline, we developed four individual subsystem models, based on which a set of variables from each subsystem that were predictive of intelligibility decline were derived (Aim 1); we then established one multi-subsystem model of intelligibility that predicted the contribution of the variables from each subsystem to the overall decline in intelligibility (Aim 2). Because there was more data on the higher end of intelligibility distribution than on the lower end (e.g., < 40%), we only modeled intelligibility within the range of 40~100% to minimize the effect of outliers in the lower part of the total range. In the individual subsystem models, intelligibility was modeled as a function of the principal components of each subsystem using linear and nonlinear mixed effects (LME/NLME) modeling approaches [30–32]. In the multi-subsystem model, intelligibility was modeled as a function of selective predictors from the articulatory, resonatory, phonatory, and respiratory subsystems based on a stepwise regression.

#### Individual subsystem models of intelligibility decline

As shown in the second panel of the flow chart in Fig 1, each subsystem model was determined in multiple steps. First, both an LME model (*fitlme*, MATLAB R2013b) and an NLME model (*nlmefit*, MATLAB R2013b) were applied to predict intelligibility using the PCs of each subsystem as predictors, while accounted for inter-subject variability. A linearity test (*coefTest*, MATLAB R2013b) was then applied to the LME model to test the linearity assumption. If the linearity assumption was violated (*p* > = .05), the NLME model was selected. If the linearity assumption was not violated (*p* < .05), the LME model was compared with the NLME model using the Bayesian information criterion (BIC) and the model with a smaller value of BIC was selected as the optimal fit model. The procedures for determining the form of the LME/NLME model are elaborated on in S1 Text. In brief, if there was a single PC in the model, intelligibility was modeled as a linear or bi-phasic function of this PC, depending on which model provided a better fit. If there were two PCs in the model, intelligibility could be either linearly or nonlinearly correlated with each PC, resulting in three candidate forms–one LME model and two NLME models. The Bayesian information criterion selected the one with the best fit as the optimal model out of all candidate models, including all NLME model(s) that converged and the LME model. If none of the NLME models converged, then the LME model was selected. Based on the selection of the best-fitting model for each subsystem, a set of subsystem predictors was generated.

#### Data imputation

Due to the high dropout rates, each predictor had a number of cases with missing data. These cases with missing data are by default discarded in statistical models, resulting in reduced sample size and imbalance of the data, which might potentially bias the results. To increase the sample size and reduce the imbalance in the data, we imputed each subsystem predictor based on its relation to intelligibility represented by each subsystem model. Specifically, we estimated the missing values of the subsystem predictor given the observed values of intelligibility by solving the equation in the subsystem model. If there was more than one predictor with overlapped cases of missing data in the subsystem model, then the problem was considered as underdetermined, and imputation was not done. All remaining cases with missing data were imputed for each subsystem predictor.

#### Multi-subsystem model of intelligibility decline

Following the steps in the third panel of the flow chart in Fig 1, the multi-subsystem model of intelligibility decline was determined as a function of all four subsystems. To select model covariates, the imputed subsystem predictors were first tested for multicollinearity (*vif*, R 3.0.1) based on the variance inflation factor (*VIF*). All subsystem predictors with a cutoff *VIF* < 3 were selected and applied as the inputs to a stepwise regression (*step*, R 3.0.1), which used the Akaike information criterion (AIC) to select a subset of predictors with minimal inter-correlations that comprised the best-fitting model of intelligibility.

### Impacts of cognitive-linguistic deficits and general respiratory status on speech performance

Although motor symptoms predominate, 10% of patients with ALS exhibit symptoms of frontotemporal dementia (FTD) and up to 50% show signs of cognitive-linguistic deficits [33], which could impact speech intelligibility and some of the subsystem measures (e.g., speech pauses). Moreover, in addition to the respiratory component of speech intelligibility assessed in the study, the general respiratory status could also impact the speech performance of patients with ALS.

Because our goal was to understand the changes in bulbar motor performance and their effects on speech intelligibility, the potential confounding effects of cognitive-linguistic deficits and general respiratory status on speech performance needed to be minimized on the intelligibility model. To assess the impact of general respiratory status on speech performance, we applied a linear mixed effects model (*lmer*, R 3.2.3) to examine the relation between speech intelligibility and the % Forced Vital Capacity (%FVC), while controlling for the effect of bulbar motor impairment, which was represented by the bulbar subscore on the ALSFRS-R. The model showed no significant correlation between %FVC and speech intelligibility, suggesting that the general respiratory status did not have a significant impact on the speech performance of the participants in this study.

To assess the impact of cognitive-linguistic deficits, we examined the relation between speech pause duration, which was demonstrated to be sensitive to both motor impairments and cognitive-linguistic deficits [33], and articulation rate, which is only affected by motor impairments [33], while controlling for respiratory status. A linear mixed effects model (*lmer*, R 3.2.3) was applied, which showed a significant correlation (*p<0*.*0001*) between speech pause duration and articulation rate with an *R*^*2*^ of 0.7. This strong correlation indicated that the bulk of the variance in speech pause duration was accounted for by articulation rate, suggesting that motor impairments were the primary deficits in the participants of this study. Moreover, we found no significant correlation between the residuals of the LME model above and speech intelligibility, suggesting that cognitive-linguistic deficits did not have a significant impact on speech intelligibility.

## Results

### Speech intelligibility

The SIT intelligibility scores ranged from 2.73% to 100% (M = 87.54%, SD = 22.70%) across subjects and recording sessions. The average speech intelligibility scores at the initial and final sessions were 95.05% (SD = 8.63%) and 75.20% (SD = 31.15%), respectively. The average intelligibility drop between the first and last sessions across subjects was 19.86% (SD = 27.11%).

### Principal components of speech subsystems

Table 1 lists the principal components that accounted for the bulk of variance for each subsystem along with the key composites of each principal component and their corresponding weights. Two principal components (*PCart1*, *PCart2*) accounted for over 96% of the variance in the articulatory subsystem. The measures that comprised these principal components included the maximum and minimum velocities of the composite movement of lower lip and jaw, the maximum and minimum velocities of lip opening during “Buy Bobby a puppy”, and the number of syllable repetitions produced on one breath in the AMR task. Two principal components (*PCreso1*, *PCreso2*) accounted for over 99% of the variance in the resonatory subsystem. The key measures that comprised the resonatory principal components were the peak nasal flow during /pi/ and the average nasalance score during “Buy Bobby a puppy.” One principal component (*PCresp1*) accounted for over 95% of the variance in the respiratory subsystem. The key composites of the respiratory principal component, as listed in Table 1, corresponded to the pausing pattern (i.e., number of pauses, % pause time, and pause duration) during the reading of a passage.

**Table 1:** The key variables and the corresponding weights that comprise the principal components of each speech subsystem.

Principal components	Key variables	Weights
PCart1	BBP_MaxVel_LL+JAW	0.41
	BBP_MaxVel_UL-LL	0.56
	BBP_MinVel_LL+JAW	-0.40
	BBP_MinVel_UL-LL	-0.56
PCart2	Reps_AMR	0.99
PCreso1	NasalFlow_Pi	-1.00
PCreso2	Naso_BBP	-1.00
PCphon1	Max_F0	0.97
PCresp1	Pause_Event	-0.51
	%Pause	-0.76
	Pause_Duration	-0.40

*Notes*.

BBP_MaxVel_LL+JAW = Maximum velocity of the composite movement of lower lip and jaw during “Buy Bobby a puppy.”

BBP_MaxVel_UL-LL = Maximum velocity of lip opening during “Buy Bobby a puppy.”

BBP_MinVel_LL+JAW = Minimum velocity of the composite movement of lower lip and jaw during “Buy Bobby a puppy.”

BBP_MinVel_UL-LL = Minimum velocity of lip opening during “Buy Bobby a puppy.”

Reps_AMR = Number of syllable repetitions during the AMR test

NasalFlow_Pi = Peak nasal airflow during /pi/

Naso_BBP = Median nasalance in “Buy Bobby a puppy.”

Max_F0 = Maximum fundamental frequency during a high pitch task

%Pause = Percentage of pause time during Bamboo passage

Pause_Event = Number of pauses during Bamboo passage reading

Pause_Duration = Total duration of pauses during Bamboo passage reading

For the phonatory subsystem, the first and second principal components (*PCphon1*, *PCphon2*) jointly accounted for 100% of the variance because only two pre-screened phonatory variables (i.e., maximum F0 and average laryngeal airway resistance) were subjected to PCA. However, the key measure that comprised *PCphon2* (i.e., average laryngeal airway resistance) was only available for a relatively small number of participants, which limited the statistical power of the phonatory subsystem-based intelligibility model. To determine whether *PCphon2* must be included as a predictor of intelligibility, we conducted a likelihood ratio test to compare an LME model of intelligibility with only *PCphon1* as a predictor and another LME model with both *PCphon1* and *PCphon2* as predictors. We found no statistical difference between the two models (*p* = 0.19), so *PCphon2* was dropped from the analysis. Meanwhile, because the average laryngeal airway resistance only had a minor effect on *PCphon1*, we replaced the missing values of this variable with zeros and updated *PCphon1* to serve as a predictor of intelligibility, which accounted for 77.7% variance in the phonatory subsystem.

### Individual subsystem models of intelligibility decline

**Fig 2:**
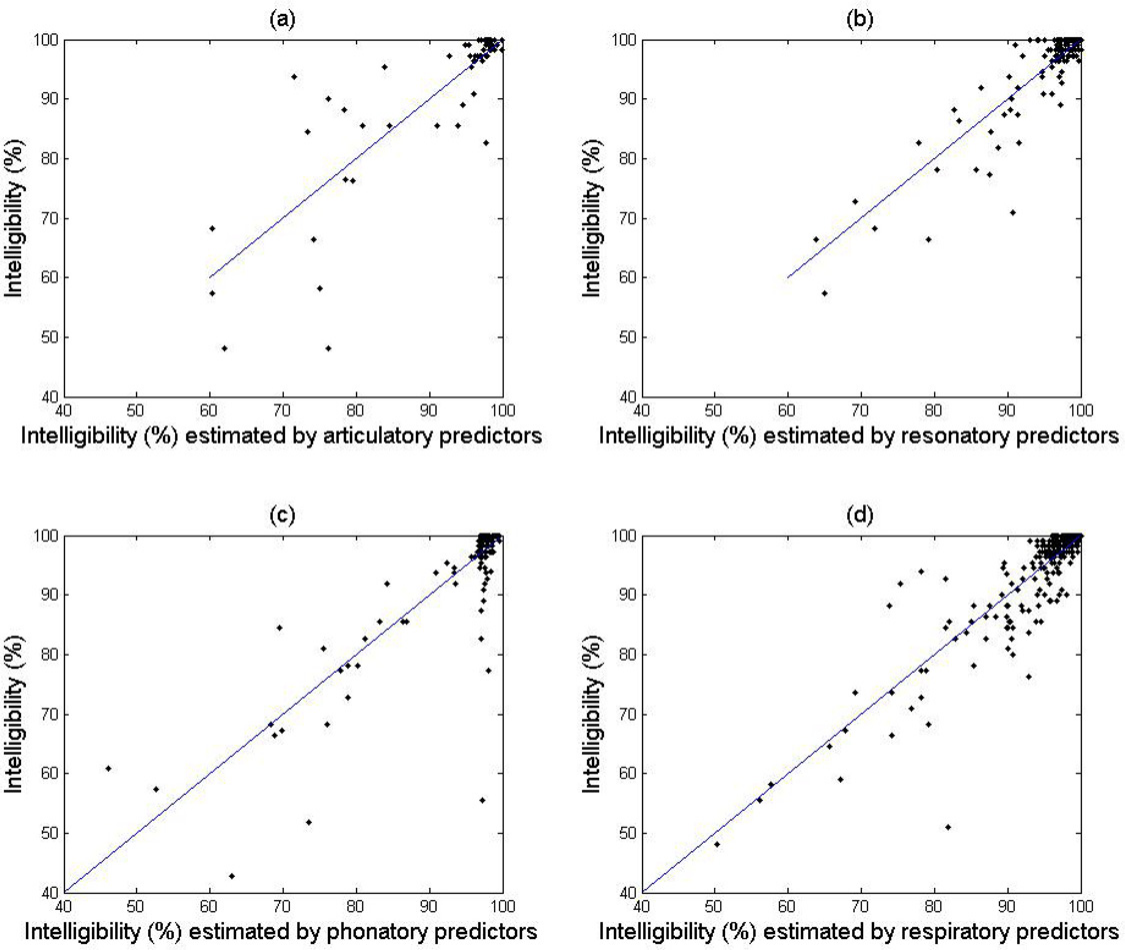
Scatter plots of intelligibility against the estimated values of intelligibility based on each individual subsystem model.

Statistical results of the intelligibility model as a function of each subsystem are shown in Table 2. For the articulatory subsystem, the NLME model with a linear term of *PCart1* and two nonlinear terms of *PCart2*, which corresponded to the early and late phases of the disease, provided a better fit than did the LME model (BIC[NLME] = 461.8, BIC[LME] = 486.1). Thus the NLME model was selected to model intelligibility as a nonlinear function of the articulatory PCs. For the resonatory subsystem, all candidate NLME models failed to converge, so the LME model was selected as the best fit to model intelligibility as a linear function of the resonatory PCs. For the phonatory subsystem, because the linearity assumption was violated (linearity test with *p* = 0.145), the NLME model with two nonlinear terms of *PCphon1*, corresponding to the early and late phases of the disease, was selected as the optimal model of intelligibility. For the respiratory subsystem, the NLME model with two nonlinear terms of *PCresp1*, which corresponded to the early and late phases of the disease, provided a better fit than did the LME model (BIC[NLME] = 1934.8, BIC[LME] = 2086). Therefore, the NLME model was selected to model intelligibility as a nonlinear function of the respiratory PC. According to the *R*^*2*^ values in Table 2 and the scatter plots in Fig 2, all of the subsystem-based models show relatively good fits.

**Table 2:** Individual subsystem models of intelligibility.

Subsystem	Fixed effects of subsystem-based model of intelligibility	R^2^
Articulatory	93.92+0.011*PCart1+0.015*max(PCart2-31.45,0)−1.4*max(31.45-PCart2,0)	0.71
Resonatory	98.62+0.0022*PCreso1+0.19*PCreso2	0.80
Phonatory	96.84+0.0047*max(PCphon1-280.07,0)−0.21*max(280.07-PCphon1,0)	0.72
Respiratory	95.22+0.13*max(PCresp1+36.43,0)– 1.56*max(-36.43-PCresp1,0)	0.80

*Note*. The bold parts are subsystem predictors of intelligibility.

According to the form of the articulatory subsystem-based model, (1) intelligibility declined at a constant rate of 0.011% as *PCart1* decreased; and (2) intelligibility declined at different rates in two phases as *PCart2* decreased. The transition of the two phases was determined by a threshold of *PCart2* at 31.45: when *PCart2* > 31.45, intelligibility declined at a slower rate of 0.015% as *PCart2* decreased; and when *PCart2* < 31.45, intelligibility declined at a faster rate of 1.4% as *PCart2* decreased.

The form of the resonatory subsystem-based model suggested that (1) intelligibility declined at a constant rate of 0.0022% as *PCreso1* decreased; and (2) intelligibility declined at a constant rate of 0.19% as *PCreso2* decreased.

The form of the phonatory subsystem-based model suggested that intelligibility declined at different rates in two phases as *PCphon1* decreased: (1) when *PCphon1* > 280.07, intelligibility declined at a slower rate of 0.0047% as *PCphon1* decreased; and (2) when *PCphon1* < 280.07, intelligibility declined at a faster rate of 0.21% as *PCphon1* decreased.

The form of the respiratory subsystem-based model suggested that intelligibility declined at different rates in two phases as *PCresp1* decreased: (1) when *PCresp1* > -36.43, intelligibility declined at a slower rate of 0.13% as *PCresp1* decreased; and (2) when *PCresp1* < -36.43, intelligibility declined at a faster rate of 1.56% as *PCresp1* decreased.

Before applying the subsystem predictors shown in Table 2 to the multi-subsystem model, data imputation was performed. Specifically, 6% of the data were imputed in *PCart1*; 27% of the data were imputed in *max(PCart2-31*.*45*,*0)* and *max(31*.*45-PCart2*,*0)*; 8% of the data were imputed in *PCreso1* and 4% of the data were imputed in *PCreso2*; 61% of the data were imputed in *max(PCphon1-280*.*07*,*0)* and *max(280*.*07-PCphon1*,*0)*; 27% of the data were imputed in *max(PCresp1+36*.*43*,*0)* and *max(-36*.*43-PCresp1*,*0)*.

### Multi-subsystem model of intelligibility decline

Nine subsystem predictors (3 articulatory, 2 resonatory, 2 phonatory, and 2 respiratory predictors as bolded in Table 2) were subjected to the collinearity test. Among all predictors, one phonatory predictor (i.e., *max[280*.*07-PCphon1*,*0]*) was identified to be highly correlated with the other 8 predictors (*VIF* = 13.36), so this predictor was discarded from further modeling. Among the remaining 8 predictors, one respiratory predictor (i.e., *max[-36*.*43-PCresp1*,*0]*) and one articulatory predictor (i.e., *max[31*.*45-PCart2*,*0]*) were identified to be correlated with each other with an *R*^*2*^ greater than 0.6 (*VIF* > 3). To determine which of these two predictors should be discarded, we compared two stepwise regressions. In the first stepwise regression, all of the subsystem predictors except *max[280*.*07-PCphon1*,*0]* and *max[-36*.*43-PCresp1*,*0]* were applied as the covariates to predict intelligibility. Five predictors (i.e., *PCart1*, *max[31*.*45-PCart2*,*0]*, *PCreso1*, *max[PCphon1-280*.*07*,*0]*, *max[PCresp1+36*,*43*,*0]*) were selected by the stepwise regression, which combined accounted for 95.6% of the variance in the overall decline of intelligibility. In this model, the articulatory subsystem showed the most substantial contribution (57.7%) to intelligibility decline; the resonatory subsystem showed a moderate contribution (22.7%); and the phonatory and respiratory subsystems showed minor contributions (8.3%, 7.2%, respectively) to intelligibility decline. In the second stepwise regression, all of the subsystem predictors except *max[280*.*07-PCphon1*,*0]* and *max[31*.*45-PCart2*,*0]* were applied as the covariates to predict intelligibility. Five predictors (i.e., *max[-36*.*43-PCresp1*,*0]*, *max[PCart2-31*.*45*,*0]*, *PCreso1*, *PCreso2*, *max[PCphon1-280*.*07*,*0]*) were selected by the stepwise regression, which jointly accounted for 79.1% of the variance in the overall intelligibility decline. By comparing the two stepwise regressions, we selected the one with a greater *R*^*2*^ (i.e., the first one) to comprise the multi-subsystem model of intelligibility decline. The corresponding subsystem predictors, model parameters, and statistics are listed in Table 3.

**Table 3:** Parameters and statistics of the multi-subsystem model of intelligibility decline.

Subsystem	Predictor	Beta coefficient	p-value	Independent contribution
Articulatory	PCart1	0.0043	0.046*	0.577
	max(31.45-PCart2,0)	-1.27	< .001*	
Resonatory	PCreso1	0.008	< .001*	0.227
Phonatory	max(PCphon1-280.07,0)	0.0031	< .001*	0.083
Respiratory	max(PCresp1+36.43,0)	0.033	0.069	0.072

*Notes*. The multi-subsystem model of intelligibility decline is in the following form, which corresponds to an *R*^*2*^ of 0.956

Intelligibility ≅ 96.39 + 0.0043*PCart1-1.27*max(31.45-PCart2,0)+0.008*PCreso1 + 0.0031*max(PCphon1-280.07,0) + 0.033*max(PCresp1+36.43,0)

According to the multi-subsystem model, over the course of the disease, intelligibility declined at a rate of 0.0043% as *PCart1* decreased and at a rate of 0.008% as *PCreso1* decreased. When *PCphon1*, *PCresp1*, and *PCart2* decreased, intelligibility declined at various rates during different stages. Specifically, when *PCphon1* was > 280.07, intelligibility declined at a rate of 0.0031% as *PCphon1* decreased. When *PCresp1* was > -36.43, intelligibility declined at a rate of 0.033% as *PCresp1* decreased. When *PCart2* was < 31.45, intelligibility declined at a rate of 1.27% as *PCart2* decreased.

## Discussion

A data driven approach was used for identifying a set of speech subsystem variables that predicted speech intelligibility decline secondary to ALS. The subsystem variables were extracted from a large, comprehensive set of instrumental measures (i.e., acoustic, aerodynamic, or kinematic) of articulatory, resonatory, phonatory, and respiratory functions. From this large set, a small subset of variables was used to generate an explanatory model of intelligibility decline based on the relative contribution of the selected speech subsystem predictors—2 articulatory (i.e., slowed lip and jaw movement, reduced AMRs), 1 resonatory (i.e., increased nasal airflow during stop consonants), 1 phonatory (i.e., reduced maximum F0), and 1 respiratory (i.e., increased speech pauses). These predictors jointly explained 95.6% of the variance in the overall decline of intelligibility. Among all subsystems, the articulatory subsystem showed the most substantial contribution (57.7%) to intelligibility decline; the resonatory subsystem showed a moderate contribution (22.7%); and the phonatory and respiratory subsystems only accounted for a small amount of the variance in intelligibility loss (8.3%, 7.2%, respectively). This explanatory model of speech intelligibility decline enhances our understanding of the physiologic mechanism underlying speech loss due to neurologic impairments, which can potentially improve speech motor assessments and help clinicians to identify treatment targets.

### Multi-subsystem model of speech intelligibility decline in ALS

Because speech is supported by the coordinated actions of multiple subsystems (i.e., articulatory, resonatory, phonatory, and respiratory), models of speech intelligibility will need to account for the individual and collective contributions from all of the speech subsystems. Compared to the models of speech intelligibility in previous studies [5,6], our model accounted for a larger portion of variance in intelligibility decline (i.e., 95.6%). The improvement might have been due to several factors.

First, our subsystem measures were instrumentation based, which provided objective indices of subsystem functions that were not affected by listener effects compared to subjective measures such as the auditory-perceptual features used in De Bodt et al. [5]. Second, we modeled both the cross-sectional and longitudinal aspects of intelligibility decline in persons with varying severities of ALS over a relatively wide time span. Unlike the linear regression models used in previous studies, the mixed-effects models parceled out the variations of intelligibility due to longitudinal decline relative to those due to cross-subject differences, which may have minimized the potential confounding effect of the large inter-subject variation in disease presentation and severity. Third, unlike prior studies, we modeled two phases of intelligibility decline using a nonlinear approach. Modeling the bi-phasic aspect of intelligibility decline is important for a disease like ALS because the transition across the two phases might indicate critical changes in subsystem functions during the disease progression, which could potentially be used to assist disease monitoring and clinical intervention. Fourth, the data driven approach effectively identified a subset of variables that represent the principal characteristics of each subsystem, which were used to predict intelligibility without a priori assumptions about the underlying relation between intelligibility and subsystem measures.

Among the five predictors selected by the stepwise regression, *PCart1*, which represented the velocity of the composite movement of lower lip and jaw and the velocity of lip opening, and *PCreso1*, which corresponded to the nasal airflow during oral consonants, accounted for the bulk of variance in the longitudinal decline of intelligibility. This finding is consistent with prior studies of ALS and dysarthria. For example, De Bodt et al. [5] and Lee et al. [6] both found that the articulatory subsystem contributed to the largest portion of variance (i.e., 47% and 58%, respectively) in intelligibility in various types of dysarthria, which is consistent with our finding of a predominant contribution (57.7%) of articulatory impairments to intelligibility decline. Kelhetter [34] examined the nasal air pressure in oral consonants produced by three individuals with ALS and found that increased nasal air leakage was only moderately associated with reduced intelligibility due to ALS. This result is consistent with our finding of a moderate contribution (22.7%) of increased nasal airflow during oral consonants (*PCreso1*) to intelligibility decline.

### Key variables predictive of intelligibility decline

The data-driven approach identified a small number of features for each subsystem that were highly predictive of intelligibility decline from a large multi-factorial dataset. For the articulatory subsystem, two prominent features were identified–slowed lip and jaw movements (*PCart1*), and slowed AMRs (*PCart2*). These articulatory features were expected in response to bulbar motor neuron deterioration. As the speed of lip and jaw slowed (i.e., *PCart1* decreased), intelligibility declined at a constant rate (see Table 2), suggesting that the speed of lip and jaw movements might serve as effective predictors of impending declines in speech intelligibility. The slowing of articulatory movements is expected to degrade speech intelligibly when it coincides with decreases in the distinctiveness among consonants and among vowels [35–37]. Yunusova et al. [19] also reported that reductions in lip and jaw movement speed were associated with a precipitous drop in intelligibility. Because jaw or lip function appear to be less affected by motor neuron degeneration than is lingual function [11,38], we anticipate that adding tongue data to the model, which is the focus of our on-going data collection and analysis, would only strengthen the association between articulatory subsystem performance and speech intelligibility–although changes in jaw function may be indicative of the tongue impairment [39].

Among the resonatory subsystem variables, the best predictors of intelligibility decline included increases in the nasal airflow during oral consonants (*PCreso1*) and increases in nasalance in a sentence with oral consonants (*PCreso2*), which are secondary to velopharyngeal inadequacy. Intelligibility declined at a constant rate (see Table 2) as velopharyngeal inadequacy increased. Prior research on hypernasality has established its global impact on speech acoustics (e.g., reduced oral acoustic energy, altered formant structures, increased nasal resonance), which combined have an impact on the acoustic distinctiveness of phonemes [40–41]. Although information about how ALS affects velopharyngeal function is scant, even modest increases in nasality are expected to reduce speech intelligibility particularly when it coincides with articulatory imprecision. Delorey et al. [14] and Kelhetter [34] assessed the velopharyngeal function in persons with ALS using aerodynamic and acoustic measurements, and observed increased nasal airflow/pressure and greater nasalance scores (i.e., by about 12% in the subgroup with hypernasality compared to the subgroup with nonbulbar impairment). Ball et al. [27], anecdotally, reported that speech was most unintelligible in persons with ALS who were unable to maintain sufficient velopharyngeal closure during pressure consonants.

The best phonatory predictor of intelligibility loss was maximum F0 during a “high pitch” task (*PCphon1*). The upper range of pitch is likely to be compromised secondary to vocal fold weakness and/or spasticity [17]. Poor laryngeal control can contribute to phonetic contrast errors (e.g., voicing errors) and abnormal prosodic patterns that characterize the dysarthria associated with ALS [2]. Among various phonatory variables, Kent et al. [1] identified F0 range as one of the most indicative acoustic correlates of dysarthric intelligibility impairment. Bunton et al. [42] subsequently found that the flattening of F0 contour had detrimental effects on intelligibility.

The best respiratory predictor of intelligibility loss was speech pausing pattern (*PCresp1*). The increase in speech pauses was most likely due to respiratory muscle weakness [43]. As the number of pauses in speech increased (i.e., *PCresp1* decreased), intelligibility declined in two phases, with the second phase declining more rapidly than the first phase. Additional work is required to determine the mechanisms that give rise to these different phases of decline. One possibility is that during the slow phase of intelligibility decline, pausing may be adaptive for maximizing speech intelligibility; whereas during the fast phase of intelligibility decline, the number of pauses is increased because of the primary disease effects on respiratory musculature.

### Maximum performance tasks as early indicators of subclinical bulbar decline

The findings from the articulatory and phonatory-based models (see Table 2) suggest that the extent to which maximum performance tasks are predictive of intelligibility decline varies depending on the stage of the disease. During the early stage of the disease (i.e., when the number of syllables produced on one breath in the AMR task was above 32 and maximum F0 was above 280 Hz), rapid declines in AMR performance and maximum F0 occurred while speech remained intelligible. Consequently, the association between these variables and speech outcomes was weak during this stage, which may explain why variables such as the rate of speech muscle contraction or even oromotor strength have been viewed as ineffective predictors of speech outcomes [11,44,45]. The observation that these measures of maximum performance tasks change during the early stages of the disease, however, suggest they are useful markers of early bulbar involvement and, potentially, early indicators of impending speech loss [3,19].

Speech intelligibility started to decline precipitously only after the number of syllables produced on one breath in the AMR task dropped below 32 and maximum F0 dropped below 280 Hz. During this stage of the disease, declines in AMRs and maximum F0 were strongly associated with the decline in speech intelligibility. These findings raise the possibility that speech intelligibility starts to decline only after speech muscle functions degenerated to levels that are required to generate speech. Because tongue and lip muscles only generate 10 to 30% of their maximal forces during speech, and the mandibular system only generates less than 2% of the maximal muscle contraction for speech, DePaul et al. [11] suggested that the weakness of tongue, jaw and lip muscles during the early stage of ALS is of little consequence to speech intelligibility. Similarly, in a study of speech and voice decline due to ALS, Rosenfield et al. [46] concluded that clinically significant impairments of speech intelligibility occurs only after laryngeal impairment reaches a critical level.

Prior studies have primarily used alternating motion rates rather than the number of repetitions produced during the AMR task to assess speech motor function [12,13,15,19,47]. In this study, alternating motion rate was found to be correlated with the velocities of lip and jaw movements and was thus eliminated by the data-driven approach from the predictors of intelligibility decline. Instead, the number of syllables produced during the AMR task was proven to contribute to a substantial portion of variance in intelligibility when it is combined with the velocities of lip and jaw movements (see Table 2) as predictors of intelligibility decline. Yet, the cutoff below 32 syllables, which was identified as the threshold that marked the onset of precipitous intelligibility decline, should be interpreted cautiously until additional research is conducted to determine the expected variation of this measure across a large sample of healthy controls and persons with ALS.

### Interdependencies among speech subsystem variables—a challenge to the modeling of speech intelligibility decline

Although the subsystem variables in this study are assumed to assess the isolated status of the targeted speech subsystem, in practice, some of the variables are expected to covary because of acoustic, aerodynamic, or biomechanic dependencies among the speech subsystems. For example, speech pausing pattern (*PCresp1*), which was used to indicate respiratory function in this study, is not only affected by respiratory impairment but also by articulatory and laryngeal weakness. Inefficiency in airflow management at the laryngeal and oropharyngeal levels necessitated more frequent inspirations. Therefore, the increase in speech pauses might reflect an interaction between the respiratory and articulatory (and/or phonatory) subsystem impairments, which could all result in declines in intelligibility. Similarly, nasal airflow is impacted not only by the velopharyngeal status but also by oropharyngeal articulation [48]; increased nasal airflow might be the outcome of either increased velopharyngeal inadequacy, or slowed lip and jaw movements or a combination of the two deficits. Interdependence among subsystem measures is a common challenge for both instrumental and subjective assessments of speech motor performance [6]. To address this challenge, the stepwise regression identified a subset of subsystem variables with minimal interdependences, which combined predicted a substantial proportion (i.e., 95.6%) of variance in intelligibility. Some subsystem variables that were highly predictive of intelligibility in the individual subsystem models were excluded from the multi-subsystem model because of their strong correlations with other subsystem variables. For example, in the individual subsystem models, AMR (*PCart2*), maximum F0 (*PCphon1*), and speech pauses (*PCresp1*) were all identified to be predictive of the rapid phase of intelligibility decline. When considering the interdependence of these variables, we found that although AMR was intended to assess orofacial deficits, it was also affected by respiratory and phonatory impairments. Impairments to the respiratory function could lead to both decreases in AMR repetitions and increases in speech pauses, resulting in a covariation of *PCart2* and *PCresp1*. We tested the effects of *PCart2* and *PCresp1* on the rapid phase of intelligibility decline when combined with other subsystem predictors and found the model with *PCart2* provided a better prediction of the overall decline in intelligibility. As a result, *PCresp1* was not selected by the stepwise regression to serve as a predictor of the multi-subsystem model. Similarly, laryngeal deficits could lead to both voice disturbances such as reductions in maximum F0 and increased voice onset time in the AMR task. As a result, maximum F0 was found to be highly correlated with AMR repetitions (*VIF*>10), so *PCphon1* was not selected as a predictor of the multi-subsystem model.

### Challenges with missing data

The goal of this study was to screen a great variety of speech subsystem variables (N = 58) in a relatively large number of persons with ALS (66 participants) and across multiple sessions (average = 7 sessions for each participant). When recording such a large number of variables for each session, missing cells are expected due to either equipment failure or increasing fatigue within a session as the disease progresses [49,50]. To minimize the potential biasing effect of missing data within a subsystem, we imputed the missing data in each subsystem predictor based on its relation to intelligibility decline, which allowed for the inclusion of data from 126 sessions.

Although the imputation treatment was effective for maximizing the amount of data that could be included in the model, the approach has several potential shortcomings. First, the missing data across different participants was imputed based on the group pattern, which may have attenuated inter-subject variability and, in turn, inflated the *R*^*2*^ of the multi-subsystem model. In addition, the imputation was not effective when there was more than one predictor from the same subsystem with overlapping cases of missing data. Therefore, for some subsystem predictors (e.g., *PCart1*, *PCreso1*, *PCreso2*), only a small portion of missing data could be imputed.

### Clinical Implications

The results of this study suggest that it is possible to anticipate both the onset and rate of critical speech changes occurred during different stages of the disease, by monitoring subsystem functions. Based on the relation between the subsystem variables identified by the data-driven approach and speech intelligibility, we propose a protocol combining AMR and maximum phonation tasks, which can be implemented in a clinical setting for speech subsystem assessment.

As has been discussed, persons with ALS show declines in their AMR performance prior to the presence of speech intelligibility deficits. Rong et al. [51] further demonstrated that the AMR performance during the early stages of ALS was predictive of the later decline of speech intelligibility. Specifically, approximately 6 months prior to the presence of clinical speech symptoms (e.g., speech intelligibility deficits or slowing of speaking rate), the AMR already showed an average difference of 1.4 syllable per second between individuals with a later fast progression rate of speech decline (i.e., fast bulbar disease progressors) and those with a relatively slow progression rate (i.e., slow bulbar disease progressors). This difference of AMR between fast and slow progressors increases over time as the disease progresses. By monitoring the AMR performance, clinicians can stratify fast progressors at relatively early stages of the disease for clinical trials, which can potentially shorten the duration and reduce the cost of the trials.

The maximum phonation task provides information on the timing of clinically significant speech impairment. Our finding suggests that a drop in maximum F0 below 280 Hz (see Table 2) indicated that bulbar muscle impairments exceeded a critical level, leading to loss of speech intelligibility. This empirically-derived threshold of clinically significant speech impairment may be useful for planning the transition to augmentative assistive communication (AAC) prior to the loss of functional speech. In addition, the accurate identification of the stages of speech deterioration utilizing objective measures will only complement the currently employed standard of clinical rating scales [19].

## Conclusions and Future directions

This study shows that the longitudinal decline of speech intelligibility is primarily attributed to declines in the articulatory function, as indicated by reductions in lip and jaw movement velocities, and secondarily to declines in resonatory function, as indicated by increases in nasal airflow leakage. Future studies will focus on identifying behavioral strategies that target the articulatory and resonatory subsystems to prolong intelligible speech. Furthermore, models of speech decline at the individual level are necessary for predicting individual responses to treatments designed to improve speech intelligibility. This information would help clinicians determine the targets for clinical interventions for individuals at different stages of the disease.

## Supporting Information

S1 Table Patient characteristics. (DOCX)

S2 Table Instrumentation and data acquisition settings for measurements of speech subsystem functions. (DOCX)

S3 Table Subsystem variables that are significantly (*p* < .05) correlated with speaking rate. (DOCX)

S1 Text Determine the form of the mixed effects model of intelligibility using the principal components of each subsystem as covariates. (DOCX)

S2 Text Determine subsystem predictors of intelligibility. (DOCX)
