# Supplementary material for: Predicting Speech Intelligibility Decline in Amyotrophic Lateral Sclerosis Based on the Deterioration of Individual Speech Subsystems
Source: PLoS One. 2016 May 5;11(5):e0154971. doi: 10.1371/journal.pone.0154971 (PMC4858181; doi:10.1371/journal.pone.0154971)
Supplement: S1 Table — (DOCX) [file pone.0154971.s001.docx]

| S1 Table  *Patient characteristics* | | | | | | | | | |
| --- | --- | --- | --- | --- | --- | --- | --- | --- | --- |
| **Subject ID** | **Gender** | **Site of onset** | **Age** | **ALSFRS (Total)** | **ALSFRS (Bulbar)** | **Speech intelligibility** | **Speaking rate** | **Medical history** | **Family history** |
| 1 | M |  | 51 | 38 | 12 | 97.27 | 189.12 |  |  |
| 2 | M | B | 73 |  |  | 80 | 118.12 |  |  |
| 3 | F | B | 47 |  |  |  |  |  |  |
| 4 | M | C/L | 45 |  |  |  |  |  |  |
| 5 | M | L | 55 | 29 | 12 | 98.18 | 241.23 |  |  |
| 6 | M | C/L | 50 | 50 |  | 100 | 222.55 |  |  |
| 7 | M | C/L | 56 |  |  | 97.27 | 173.65 |  |  |
| 8 | F | C | 54 | 37 |  | 97.27 | 164.18 | COPD Asthma |  |
| 9 | F | B | 51 |  |  | 53.64 | 55.56 | Asthma | Alzheimer's Disease |
| 10 | F | B/L | 59 |  |  | 99.09 | 157.14 | Depression |  |
| 11 | F | B | 65 |  |  | 99.09 | 175.54 | Hypertension |  |
| 12 | F | B/R | 49 |  |  | 82.73 | 114.98 | Depression Hypertension |  |
| 13 | M | C | 77 |  |  | 96.36 | 163.37 | Hepatitis C- dormant |  |
| 14 | M | L | 47 |  |  | 99.09 | 218.54 |  | ALS/Other MND Other Dementia |
| 15 | M | C/L | 50 |  |  | 100 | 194.69 | Depression Asthma | Parkinson's Disease |
| 16 | F | B | 62 | 38 | 7 | 92.73 | 86.39 |  |  |
| 17 | F | C/L | 63 | 34 | 8 | 100 | 176.48 |  |  |
| 18 | F | C | 41 | 33 | 11 |  |  | Ulcerative colitis | Parkinson's Disease Alzheimer's Disease |
| 19 | M | C/L/T | 75 | 38 | 12 | 97.27 | 108.2 | Cancer Diabetes |  |
| 20 | M | C | 45 | 37 | 10 | 98.18 | 188.03 | Depression | ALS/Other MND |
| 21 | M | C | 74 | 46 | 12 | 98.18 | 150.68 |  | Alzheimer's Disease |
| 22 | F | L | 56 | 41 | 12 | 97.27 | 188.57 |  | Parkinson's Disease |
| 23 | F | B | 53 | 40 | 7 | 60.91 | 90.04 | Depression | Parkinson's Disease |
| 24 | F | B | 80 | 43 | 9 | 85.45 | 66.53 |  |  |
| 25 | F | B | 70 | 37 | 5 | 93.64 | 93.35 |  |  |
| 26 | M | B | 42 |  |  | 97.27 | 140.72 | Hypothyroidism |  |
| 27 | F | C | 59 |  |  | 100 | 126.92 |  |  |
| 28 | F | C/L | 73 |  |  | 98.21 | 201.2 | High Blood Pressure Hypothyroidism Gout of the left foot Glaucoma Hypercholesterolemia |  |
| 29 | M | L | 63 | 34 | 12 | 98.18 | 141.63 | Osteoperosis Kidney stones | ALS/Other MND |
| 30 | F | C/L | 58 | 41 | 11 | 98.18 | 150 | Uterine fibroid diagnosed in 1997 | ALS/Other MND |
| 31 | F | C/L | 64 | 45 | 12 | 100 | 144.74 | Celiac Disease Hypothyroidism |  |
| 32 | F | B | 53 | 32 | 7 | 96.36 | 134.97 |  |  |
| 33 | F | C/ L/ N | 58 |  |  | 100 | 159.04 | Osteoperosis |  |
| 34 | M | C/L | 75 |  |  | 100 | 225.26 | Spil stenosis |  |
| 35 | M | L | 46 | 33 | 12 |  |  | Hypercholesterolemia |  |
| 36 | M | B | 63 | 36 | 6 | 97.27 | 160.58 | Cancer Hypothyroidism Gout Millington melanoma |  |
| 37 | F | L | 50 | 40 | 12 | 100 | 160.59 | Diabetes High Blood Pressure | ALS/Other MND |
| 38 | M | C | 49 |  |  | 94.55 | 136.6 | Rosacea |  |
| 39 | M | L | 67 | 46 | 12 | 100 | 188.85 |  |  |
| 40 | M | C/L | 41 | 35 | 8 | 100 | 118.07 |  |  |
| 41 | M | L | 73 | 40 | 12 | 100 | 208.86 |  | Other Dementia |
| 42 | M | B | 57 | 40 | 4 | 82.73 | 95.93 | Cancer |  |
| 43 | M | B | 65 | 39 | 9 | 100 | 127.91 | Diabetes |  |
| 44 | F | C | 52 | 38 | 12 | 100 | 210.87 |  |  |
| 45 | F | L | 63 | 37 | 7 | 100 | 167.51 | Depression |  |
| 46 | M | C | 68 |  |  | 100 | 191.3 |  |  |
| 47 | M | B | 62 | 35 | 7 | 88.18 | 176.94 | Hypertension Dyslipidemia Meningtis Shingles Peripheral vascular disease |  |
| 48 | F | B | 66 | 46 | 10 | 94.55 | 89.8 | Hypertension Left inguil hernia |  |
| 49 | F | C | 68 | 30 | 12 | 97.27 | 201.22 | Hypothyroidism |  |
| 50 | F | L | 64 | 32 | 6 | 87.27 | 105.6 | Hypercholesterolemia Athsma Arthritis |  |
| 51 | M | B/C/L | 51 | 34 | 12 |  |  |  |  |
| 52 | M | C | 59 |  |  |  |  | High cholesterol Hypertension |  |
| 53 | M | C/L | 44 | 41 | 12 | 100 | 168.97 |  | Parkinson's Disease Paterl aunt had degenerative neuron disease |
| 54 | M | C/L | 64 |  |  | 100 | 117.02 | Asthma |  |
| 55 | M |  | 55 | 39 | 11 | 99.09 | 188.67 |  |  |
| 56 | M | B/C/L | 41 | 42 | 11 | 86.36 | 144.44 |  | ALS/Other MND |
| 57 | M |  | 59 | 35 | 12 | 100 | 179.44 |  |  |
| 58 | M | C | 41 | 39 | 11 | 88.18 | 146.67 | Psoriasis TB |  |
| 59 | M | N | 70 | 48 | 12 | 97.27 | 219.28 | Cancer |  |
| 60 | M | L | 51 | 33 | 10 | 100 | 165 | DM2 Asthma CAD with stent placement in 10/2005 |  |
| 61 | F |  | 54 |  |  | 100 | 190.2 |  |  |
| 62 | M | C | 40 | 39 | 10 | 98.18 | 157.89 |  |  |
| 63 | F | L | 58 | 33 | 10 | 95.45 | 169.23 |  |  |
| 64 | F | G | 51 | 31 | 11 | 100 | 251.91 | Carpal tunnel syndrome bilaterally Psoriasis | ALS/Other MND Sister diagnosed with Hereditary spastic paraparesis |
| 65 | F | C | 55 | 33 | 11 | 98.18 | 173.78 |  |  |
| 66 | M | G | 60 |  |  | 100 | 186.44 | CAD HTN Hypercholesterolemia |  |

*Notes.* For gender, M = male, F = female.

For site of onset, B = bulbar, C = cervical, L = lumbar, T = thorasic, N = neck, G = generalized. Blank cells correspond to participants with an unknown onset site.

For medical history and family history, blank cells mean there was no reported medical history and/or family history of medical conditions from the patients.

Speech intelligibility (%) and speaking rate (WPM) were obtained from the Sentence Intelligibility Test.
