# Supplementary material for: Predicting Speech Intelligibility Decline in Amyotrophic Lateral Sclerosis Based on the Deterioration of Individual Speech Subsystems
Source: PLoS One. 2016 May 5;11(5):e0154971. doi: 10.1371/journal.pone.0154971 (PMC4858181; doi:10.1371/journal.pone.0154971)
Supplement: S2 Table — (DOCX) [file pone.0154971.s002.docx]

| S2 Table  *Instrumentation and data acquisition settings for measurements of speech subsystem functions* | | | | |
| --- | --- | --- | --- | --- |
| Subsystem | Instrument | Signal | Task | Measurements |
| Articulatory | Eagle Digital System, *Motion Analysis Corp.* | Kinematic | “Buy Bobby a puppy.”  “Say /apa/ again.” | Maximum/minimum velocities of lower lip movement relative to the jaw, the composite movement of lower lip and jaw, lip opening, and jaw movement during opening and closing gestures |
|  | Compact flash recorder, Professional quality microphone, *Conutryman E6* | Acoustic | Repeat /ba/ as clear and as fast as possible on one breath (AMR test) | Number, duration, and rate of syllable repetitions |
| Resonatory | Nasometer, *Model 6400, KAYPentax* | Acoustic | “Mama made some lemon jam.”  “Buy Bobby a puppy.” | Median nasalance in the sentences |
|  | BIOPAC system, *BIOPAC System Inc.* | Aerodynamic | /ba/, /pa/, /pi/, /ma/, /mi/, “hamper” | Intraoral pressure and nasal airflow in syllables  Ratio of intraoral pressure between oral and nasal consonant pairs  Ratio of nasal airflow between oral and nasal consonant pairs  Time lag between /m/ and /p/ in “hamper” |
| Phonatory | Compact flash recorder, Professional quality microphone, *Conutryman E6* | Acoustic | “Normal” and “high pitch” phonation of /a/.  Sustain soft, normal, and loud /a-a-a/ for 3 seconds. | Maximum phonation duration  Maximum F0  Jitter  Shimmer  NHR  Average SPL of soft, normal, and loud voice  Maximum SPL |
|  | Phonatory Aerodynamic System, *KAYPentax* | Aerodynamic | Repetition of /pa-pa-pa/ five to seven times on a single breath. | Average peak air pressure  Average peak airflow  Average laryngeal airway resistance |
| Respiratory | Phonatory Aerodynamic System, *KAYPentax* | Aerodynamic | /pa/, /pi/ | Maximum subglottal pressure during /pa/ and /pi/ |
|  | Compact flash recorder, Professional quality microphone, *Conutryman E6* | Acoustic | Bamboo passage  Read /pi/ seven times. | Speech duration, total duration, and speech pausing pattern (number of pauses, pause duration, percentage of pauses) in Bamboo passage  Speech duration, pause duration, percentage of pauses in the repetitions of /pi/ |
