# Supplementary material for: Predicting Speech Intelligibility Decline in Amyotrophic Lateral Sclerosis Based on the Deterioration of Individual Speech Subsystems
Source: PLoS One. 2016 May 5;11(5):e0154971. doi: 10.1371/journal.pone.0154971 (PMC4858181; doi:10.1371/journal.pone.0154971)
Supplement: S3 Table — (DOCX) [file pone.0154971.s003.docx]

| S3 Table  *Subsystem variables that are significantly (p<.05) correlated with speaking rate* | |
| --- | --- |
| Subsystem | Variables |
| Articulatory | Maximum and minimum velocities of lip opening, lower lip movement relative to the jaw, and the composite movement of lower lip and jaw in “Buy Bobby a puppy.”  Maximum velocities of lip opening and the composite movement of lower lip and jaw in /apa/.  Number, duration and rate of the AMR test |
| Resonatory | Intraoral pressure during /ma/ and /mi/  Nasal airflow during /pi/  Ratio of nasal airflow between /pi/ and /mi/  Time lag between /m/ and /p/ in “hamper”  Median nasalance during “Buy Bobby a puppy.” |
| Phonatory | Maximum F0  Average laryngeal airway resistance |
| Respiratory | Number, duration and percentage of pauses in Bamboo passage  Duration and percentage of pauses in the repetitions of /pi/ |
