# Supplementary material for: Predicting Speech Intelligibility Decline in Amyotrophic Lateral Sclerosis Based on the Deterioration of Individual Speech Subsystems
Source: PLoS One. 2016 May 5;11(5):e0154971. doi: 10.1371/journal.pone.0154971 (PMC4858181; doi:10.1371/journal.pone.0154971)
Supplement: S1 Text — (DOCX) [file pone.0154971.s004.docx]

If there was one covariate, depending on its relation with intelligibility (linear/nonlinear), intelligibility was modeled in one of following forms:

Model 1a: Single covariate linear model – intelligibility as a linear function of a single covariate (principal component)

$$y=a+b*x \ldots(eq1a)$$

where x is the covariate, y is intelligibility, and a, b correspond to the intercept and slope, respectively. The parameters of the model to be estimated are a and b.

Model 1b: Single covariate bi-phasic model – intelligibility as a bi-phasic nonlinear function of a single covariate (principal component)


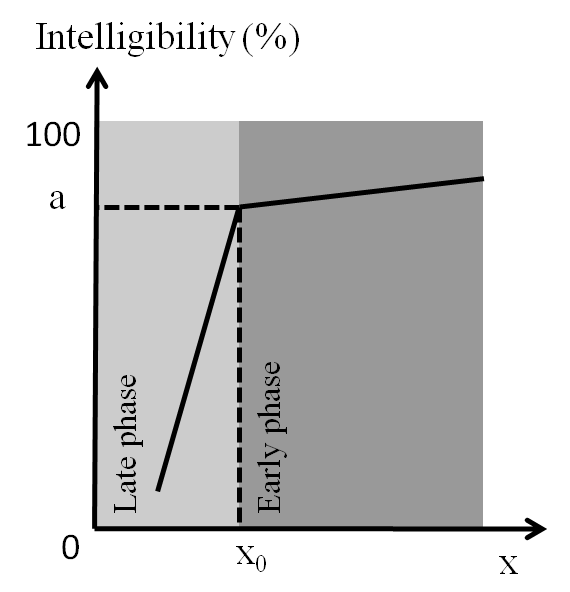
$y= a+b*\max\left( x-x_{0}, 0 \right)-c*\max\left( x_{0}-x, 0 \right)$ $=\left\{ \begin{aligned} a+b*\left( x-x_{0} \right), when x>x_{0} \\ a+c*\left( x-x_{0} \right), when x<x_{0} \end{aligned} \right. \ldots(eq1b)$

where x is the covariate, y is intelligibility, x_0_ is the transitional point of x between the early and late phases, a corresponds to the intelligibility at the transition, and b, c are slopes of the early and late decline phases, respectively. The figure on the right shows a graphical representation of the function in *eq3*. During fitting of the model, the parameters to be estimated are a, b, c, x_0_.

If there were two covariates, depending on the relation between intelligibility and each covariate (linear/nonlinear), intelligibility was modeled in one of following forms:

Model 2a: Two covariate linear model – intelligibility as a linear function of two covariates (principal components)

$$y=a+b*x_{1}+c*x_{2} \ldots(eq2a)$$

where x_1_ and x_2_ are two covariates, y is intelligibility, a is the intercept, and b, c correspond to the slopes for x_1_ and x_2_, respectively. The parameters to be estimated include a, b, and c.

Model 2b: Two covariate bi-phasic model - intelligibility as bi-phasic nonlinear functions of two covariates (principal components)

$y= a+b*\max\left( x_{1}-x_{1,0}, 0 \right)-c*\max\left( x_{1,0}-x_{1}, 0 \right)+d* \max\left( x_{2}-x_{2,0}, 0 \right)-e*\max\left( x_{2,0}-x_{2},0 \right) ... (eq2b)$

where y represents intelligibility, x_1_, x_2_ are two covariates, x_1,0_, x_2,0_ are the transitional points of x_1_, x_2_, respectively, between the two phases, b, d are slopes for x_1_, x_2_ during the early phase, and c, e are slopes for x_1_, x_2_ during the late phase. The parameters to be estimated in the model include a, b, c, d, e, x_1,0_, x_2,0_.

Model 2c: Two covariate mixed linear and bi-phasic model – intelligibility as a function of two covariates, one linear function of x_1_ and the other bi-phasic nonlinear function of x_2_

$y= a+b*x_{1}+d* \max\left( x_{2}-x_{2,0}, 0 \right)-e*max(x_{2,0}-x_{2},0) ... (eq2c)$

The parameters to be estimated in the model include a, b, d, e, x_2,0_.

Given equations *eq1a* and *eq2a*, an LME model was fitted using the *fitlme* function in MATLAB (R2013b) to predict intelligibility as a linear function of the subsystem PC(s), accounting for subject-dependent random effects on the intercept.

Given equations *eq1b, eq2b,* and *eq2c*, an NLME model was fitted using the *nlmefit* function in MATLAB (R2013b) to predict intelligibility as the pre-determined function of the subsystem PC(s), accounting for subject-dependent random effects on all model parameters except slopes.
