# Supplementary material for: Predicting Speech Intelligibility Decline in Amyotrophic Lateral Sclerosis Based on the Deterioration of Individual Speech Subsystems
Source: PLoS One. 2016 May 5;11(5):e0154971. doi: 10.1371/journal.pone.0154971 (PMC4858181; doi:10.1371/journal.pone.0154971)
Supplement: S2 Text — (DOCX) [file pone.0154971.s005.docx]

If the models defined by *eq1a* and *eq2a* were selected, the original subsystem PC(s) were used as subsystem predictors of intelligibility.

If the model defined by *eq1b* was selected, two subsystem predictors were generated, which were $\max\left( x-x_{0}, 0 \right)$ and $-\max\left( x_{0}-x, 0 \right)$ that corresponded to the early and late phases, respectively.

If the model defined by *eq2b* was selected, four subsystem predictors were generated, which included $\max\left( x_{1}-x_{1,0}, 0 \right)$ and $\max\left( x_{2}-x_{2,0}, 0 \right)$ that corresponded to the early phase, and $-max \left( x_{1,0}-x_{1}, 0 \right)$ and $-max \left( x_{2,0}-x_{2},0 \right)$ that corresponded to the late phase.

If the model defined by *eq2c* was selected, three subsystem predictors were generated, including $x_{1}$, $\max\left( x_{2}-x_{2,0}, 0 \right)$and $-max(x_{2,0}-x_{2},0)$ that corresponded to early and late phases, respectively.
